# Reconstructive Effects of Percutaneous Electrical Stimulation Combined with GGT Composite on Large Bone Defect in Rats

**DOI:** 10.1155/2013/607201

**Published:** 2013-05-29

**Authors:** Bo-Yin Yang, Tzung-Chi Huang, Yueh-Sheng Chen, Chun-Hsu Yao

**Affiliations:** ^1^Department of Chinese Medicine, China Medical University, 91 Hsueh-Shih Road, Taichung 40402, Taiwan; ^2^Department of Biomedical Imaging and Radiological Science, China Medical University, 91 Hsueh-Shih Road, Taichung 40402, Taiwan; ^3^Department of Biomedical Informatics, Asia University, 500 Liou-Feng Road, Taichung 41354, Taiwan

## Abstract

Previous studies have shown the electromagnetic stimulation improves bone remodeling and bone healing. However, the effect of percutaneous electrical stimulation (ES) was not directly explored. The purpose of this study was to evaluate effect of ES on improvement of bone repair. Twenty-four adult male *Sprague-Dawley rats* were used for cranial implantation. We used a composite comprising genipin cross-linked gelatin mixed with tricalcium phosphate (GGT). Bone defects of all rats were filled with the GGT composites, and the rats were assigned into six groups after operation. The first three groups underwent 4, 8, and 12 weeks of ES, and the anode was connected to the backward of the defect on the neck; the cathode was connected to the front of the defect on the head. Rats were under inhalation anesthesia during the stimulation. The other three groups only received inhalation anesthesia without ES, as control groups. All the rats were examined afterward at 4, 8, and 12 weeks. Radiographic examinations including X-ray and micro-CT showed the progressive bone regeneration in the both ES and non-ES groups. The amount of the newly formed bone increased with the time between implantation and examination in the ES and non-ES groups and was higher in the ES groups. Besides, the new bone growth trended on bilateral sides in ES groups and accumulated in U-shape in non-ES groups. The results indicated that ES could improve bone repair, and the effect is higher around the cathode.

## 1. Introduction

Trauma, infection, tumor resection, or skeletal abnormalities can cause bone defects of various shapes and sizes. Many methods have been applied to accelerate bone repair [1, 2]. Autografting from a tibia, fibula, iliac crest, or rib is a popular procedure but has many drawbacks such as the short supply and possible damage to the donor site. Allografting avoids these donor site problems; however, allografting has risks of immunoreaction and disease transmission [3–6]. Metal bone substitutes such as stainless steel and titanium alloys may damage the contacted normal bone and lead to inflammation from toxic ion release [1]. Bioactive ceramics, such as hydroxyapatite (HA) and tricalcium phosphate (TCP), demonstrate good biocompatibility and osteoconductive potential. Furthermore granular TCP is biodegradable [7]. In a previous study, we developed a novel composite comprising genipin cross-linked gelatin mixed with tricalcium phosphate (GGT), which is biocompatible, osteoconductive, biodegradable, and malleable [7–10]. Electromagnetic stimulation improves bone remodeling and bone healing [11–13]. Several studies have used techniques such as direct current (DC), capacitive coupling (CC), and pulsed electromagnetic field (PEMF) to stimulate bone healing. However, DC results in damage during operation; CC might cause skin hypersensitivity; and PEMF leads to the time redundant because of its time dependence [14–16]. Bone regeneration relies heavily on angiogenesis because the transportation of nutrients, oxygen, and stem cells is closely related to blood vessels [3, 17, 18]. Based on previous successful studies, the percutaneous electrical stimulation (ES) using 2 Hz and 2 mA is beneficial for angiogenesis [19, 20]. We hypothesized that the percutaneous electrical stimulation could accelerate the bone regeneration. The evaluation of percutaneous electrical stimulation for improving bone repair was investigated in present study.

## 2. Materials and Methods

### 2.1. Implant Material Preparation

Type A gelatin (Bloom number 300, Sigma Chemical Co., Saint Louis, MO, USA) with a mass of approximately 50,000–100,000 Dalton was extracted and purified from porcine skin. A homogeneous 18% gelatin solution was made by dissolving 9 g of gelatin powder in 41 mL of distilled water in a water bath at 70°C. While the gelatin solution was cooling to 50°C, a 20% genipin solution (Challenge Bioproducts Co., Taichung, Taiwan) was added to the gelatin solution to induce cross-linking reactions at a constant temperature. After stirring for 2 min, tricalcium phosphate, Ca_3_(PO_4_)_2_, ceramic particles (Merck, Germany) with grain sizes of 200–300 *μ*m were mixed into the gelatin-genipin mixture. With an inorganic/organic ratio equivalent to that in natural bone, the weight ratio of the TCP and gelatin in the composite was 3 : 1 [7, 8, 10]. The GGT composites were manually cut and shaped to a diameter of 8 mm and a thickness of 1.5 mm. All samples were frozen at −80°C for 24 h and then dried in a freeze dryer for another 24 h. 

### 2.2. Surgical Procedure

Twenty-four adult male *Sprague-Dawley rats *weighing 280–300 g were used as experimental animals for cranial implantation. The animals were kept in a stable following the national animal care guidelines. Prior to the beginning of the study, the protocol was approved by the Institutional Animal Care and Use Committee (IACUC) of China Medical University. All animals were anesthetized by the inhalation of isoflurane (Abbott, Taiwan). The head of each rat was shaved and prepared for surgery in an aseptic animal operation room. The head skin was incised in a T-shape. Next, the overlying parietal periosteum was excised. A circular (8 mm in diameter), full-thickness defect of the parietal bone was created with a drilling burr on a slow-speed dental handpiece; neither the dura nor the superior sagittal sinus was violated. All of these defects in the rats were then filled with the GGT composites. After each operation, the periosteum was closed with 5-0 vicryl, and the skin was sutured with 3-0 black silk [7, 21]. 

### 2.3. Percutaneous Electrical Stimulation Procedure

**Figure 1:**
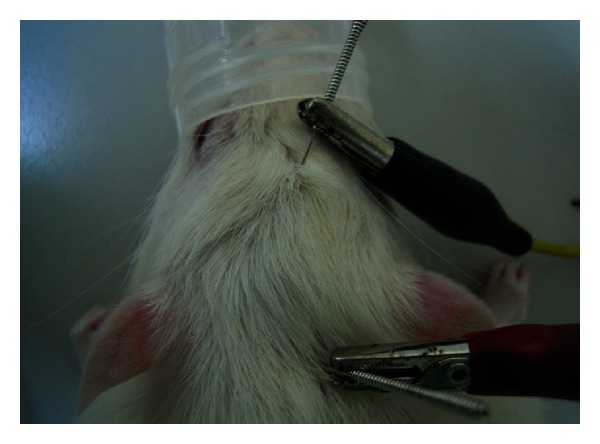
shows how the electrodes were positioned. The anode was connected to a point on the back of the neck; the cathode was connected to a front point on the head.

Percutaneous electrical stimulation using 2 Hz and 2 mA was applied in this study based on previously successful studies [19, 20]. Two points at a distance of 14 mm on the midline of the head, which were 3 mm ahead of and behind the defect, were selected for ES. After the operation, the rats were divided into six groups, with four rats each. The first three groups underwent 4, 8, and 12 weeks of percutaneous electrical stimulation (15 min/time, 3 times/week, and separated by an interval of at least one day) with stainless steel needles (0.27 mm OD, 13 mm length, Ching Ming, Taiwan) in the insertion depth of 2 mm and a stimulator (Trio 300; Ito, Tokyo, Japan). The anode was connected to a point on the back of the neck; the cathode was connected to a front point on the head, as illustrated in Figure 1. During each stimulation, the rats were under inhaled anesthesia. The other three groups, as control groups, only received inhalation anesthesia without percutaneous electrical stimulation. All the rats were examined afterward at 4, 8, and 12 weeks.

### 2.4. Harvesting, Radiomorphometry, and Histomorphometry of Tissue

The bone defect regeneration was evaluated radiographically and histologically. Using a micro-CT scanner (SkyScan-1076, Aartselaar, Belgium) and with inhalation anesthesia, each group of animals was examined 4, 8, and 12 weeks after individual percutaneous electrical stimulation. The contrast between the gray levels of the implanted material and the new bone tissue was enhanced. The volume of newly formed bone was evaluated by counting the number of voxels using ImageJ (National Institutes of Health, USA). Next, 3D images of the new bone were obtained using Amira (Visage Imaging GmbH, Berlin, Germany) to evaluate the growth trend.

Anesthetized animals were sacrificed in a carbon-dioxide-filled box 4, 8, and 12 weeks after the operation. The craniectomy sites, along with 2-3 mm of contiguous bone, were removed from each skull after the animal was sacrificed. Specimens were promptly placed into phosphate-buffered 10% formalin and prepared for further analysis. After 24 h of fixation, the specimens were radiographed in a cabinet X-ray machine (MGU 100A, Toshiba Company), using a high contrast X-ray film at 23 keV and 12.5 mA. The craniectomy site radiographs were analyzed using a semiautomatic histomorphometric method, and the regenerated bone was quantitatively evaluated as the percentage of infill area. Using an image analyzer system (Image-Pro Lite, Media Cybernetics, Silver Spring, MD, USA), a satisfactory contrast was achieved between the implanted materials and the new bone tissue by operator selection of a gray level sensitivity standard that was consistent for all treatments. The amount of newly grown bone tissue was calculated by moving a cursor on the digitizing plate, which was visible as a projection over the histological field, and this amount was expressed as a percentage of the ingrowth bone tissue in the created bone defect. 

All of the calvarial specimens were subsequently decalcified in a solution of formic acid (10%) for 1-2 weeks and then immersed in sodium sulfate overnight. The specimens were dehydrated in a graded series of ethanol and then embedded in a tissue freezing medium (OCT). Axial sections of the decalcified bone and implants (10 *μ*m thickness each) were prepared and stained with hematoxylin and eosin (H&E). To observe the relationship between the electrodes and osteoblasts or osteoclasts, longitudinal sections of other specimens (10 *μ*m thickness each) were arranged and stained with either alkaline phosphatase (ALP) stain or tartrate resistant acid phosphatase (TRAP). Photomicrographs of these sections were obtained using light microscopy.

### 2.5. Statistical Analysis

All numerical data were presented as the mean ± one standard deviation. Significant differences among the samples were evaluated using Student's *t*-test (SPSS 17.0.2). Probabilities of *P* < 0.05 were considered statistically significant.

## 3. Results

### 3.1. Gross Examination

**Figure 2:**
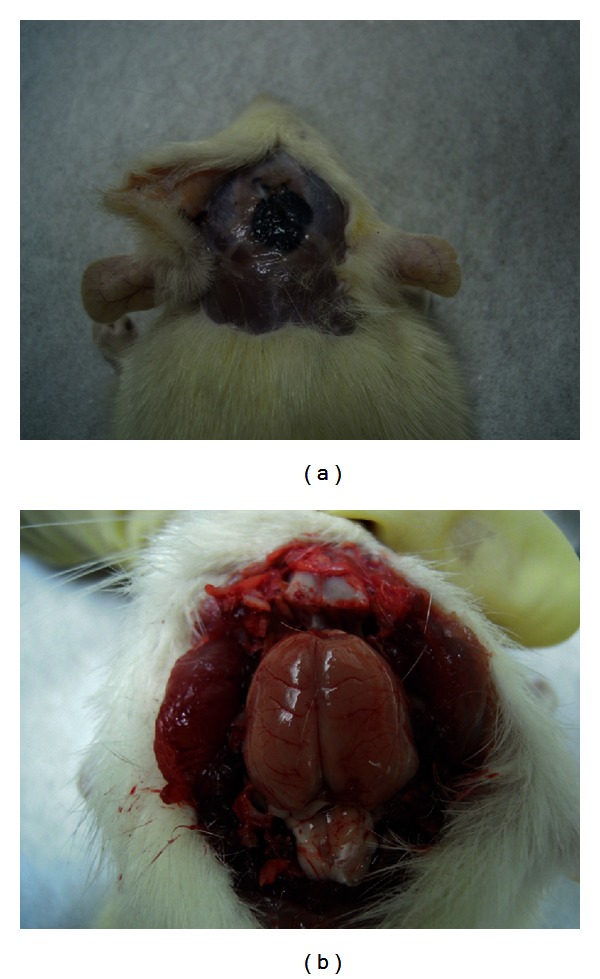
This figure represents the gross examination. (a) illustrates that there was no wound infection, scalp effusion, hematoma, festers or disturbed wound healing at the surgical site of the calvarial bone. No gaps between the GGT composite and the peripheral osseous tissues were noted, and no GGT composite was extruded. (b) shows that the brain tissues underlying the implantation site did not display any evidence of cortical inflammation, scar formation, or necrosis.

All animals in both the experimental and control groups survived for the entire experimental period without any local or general complications. There was no wound infection, scalp effusion, hematoma, festers, or disturbed wound healing at the surgical site of the calvarial bone. The results reveal that the GGT composite did not lead to histopathology or exhibit poor biocompatibility with the peripheral osseous tissues. No gaps between the GGT composite and the peripheral osseous tissues were noted, and no GGT composite was extruded (Figure 2(a)). The findings indicate that the GGT composite not only was easily molded to the calvarial bone defect without any fixation but also cohered strongly to the peripheral osseous tissues. However, cytotoxic implants may harm the underlying brain tissues because they were implanted in the calvarial bone defect and were in direct contact with brain tissue. To determine whether the brain tissue exhibited any abnormality, the calvarial bone covering implant was removed from the implantation site. The brain tissues underlying the implantation site did not display any evidence of cortical inflammation, scar formation, or necrosis (Figure 2(b)). The results revealed that the GGT composites did not cytotoxically affect the underlying brain tissue.

### 3.2. X-Ray Radiographic Analysis

**Figure 3:**
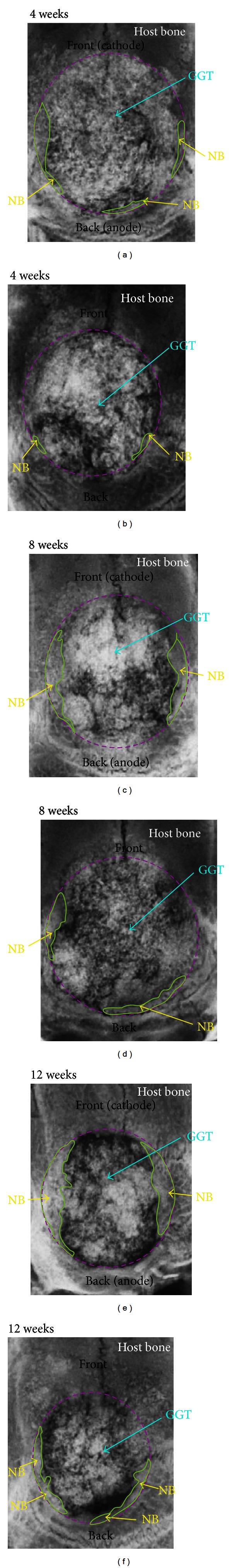
This figure shows the X-ray images. The top of each image corresponds to the front part of the rats, where the cathode is connected, and the bottom is the back part, where the anode is attached. (a), (c), and (e) display the results of the ES groups after 4, 8, and 12 weeks of bone repair, respectively. (b), (d), and (f) show the corresponding images of the control groups, in which non-ES was performed. In the ES groups, the new bone mostly formed on bilateral sides, whereas the new bone was U-shape in the non-ES groups (NB: new bone; GGT: genipin cross-linked gelatin mixed with tricalcium phosphate).

Gross examination does not determine whether the newly formed osseous tissues were completely calcified new bones. Therefore, X-ray radiographs were obtained for further analysis. The performance both with and without ES in repairing the calvarial bone defect was evaluated to determine the efficacy of ES in accelerating the healing of defective bones.  Figure 3 presents the radiographs of calvarial bone-covered implants 4, 8 and 12 weeks after the GGT composites were implanted into the calvarial bone defect. The three pictures in the left row show the ES groups, and those in the right row show the non-ES groups. In the non-ES groups, four weeks after surgery, the newly formed bone did not evidently grow into the GGT construct (Figure 3(b)); eight weeks after surgery, the implantation site had some radiopaque material in the GGT construct (Figure 3(d)); twelve weeks after surgery, the amount of radiopaque material in the GGT construct exceeded that in the radiograph obtained after 8 weeks (Figure 3(f)). The three pictures revealed that the new bone formation tended to grow in a U-shape in the non-ES groups. In the ES groups, the radiographs obtained four weeks after surgery displayed more radiopacity than those in the non-ES groups (Figure 3(a)). The radiograph obtained eight weeks after surgery showed the same pattern, and the newly formed bone accumulated on bilateral sides (Figure 3(c)). These two characteristics were more prominent in the 12-week radiograph. The newly formed bone replaced more GGT composite, and the area of the calvarial bone defect became much smaller in comparison with the 4-week and 8-week radiographs. In addition, the trend for the new bone to grow on bilateral sides was more pronounced (Figure 3(e)). 

**Figure 4:**
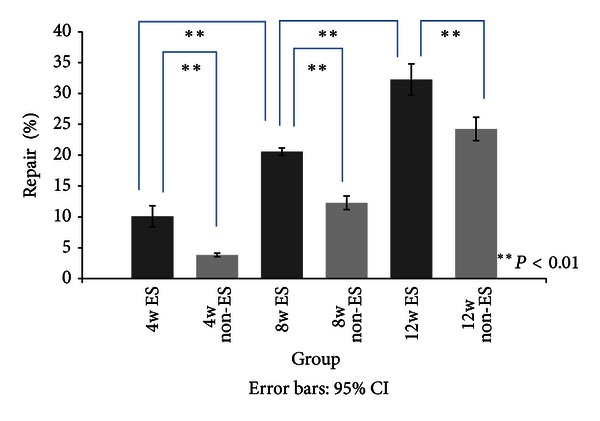
This figure shows that the percentage of bone regeneration in the rats is significantly higher in the ES groups than in the non-ES groups. The bone regenerated appreciably from 4 to 8 weeks and from 8 to 12 weeks.

The new bone formation became more obvious as the time between implantation and examination increased. Additionally, the radiographs clearly reveal that the calvarial bone defect was repaired gradually and that the GGT composite degraded progressively.  Figure 4 shows the percentage of newly formed bone to calvarial bone defect in each implantation-examination period for both groups with or without ES. The data showed augmentation in the areas of newly formed bone with time. For each implant period, the percentage of bone regeneration in the ES groups was markedly higher than that in the non-ES groups.  Figure 4 and the serial postsurgery radiographs in Figure 3 exhibit progressive wound healing. The GGT composite biodegraded, and new bone infiltrated into the implant construct over time. Although the area of newly formed bone increased with implantation-examination time in the non-ES groups, it remained lower than that in the ES groups.

### 3.3. Three-Dimensional Micro-CT Radiographic Analysis

**Figure 5:**
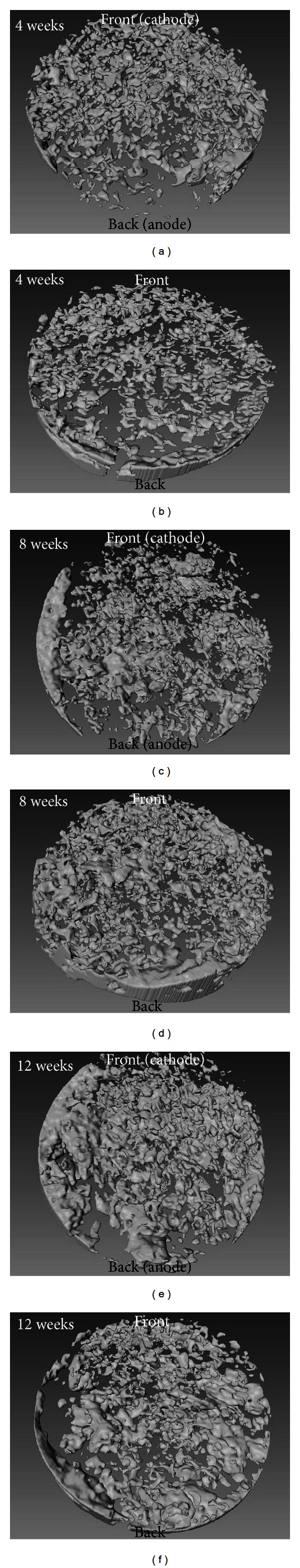
This figure shows the 3D images of the new bone. The top of each image corresponds to the front part of the rats, where the cathode is connected, and the bottom is the back part, where the anode is attached. (a), (c), and (e) display the results of the ES groups after 4, 8, and 12 weeks of bone repair, respectively. (b), (d), and (f) are the corresponding images of the control groups, in which non-ES was performed. In the ES groups, the new bone mostly formed on bilateral sides, whereas the new bone was U-shape in the non-ES groups.

A new three-dimensional (3D) method, micro-CT, was applied to evaluate the amount of new bone formation. Bone repair in both the ES and non-ES groups were evaluated.  Figure 5 displays the images of newly formed bone 4, 8, and 12 weeks after the GGT composites were implanted. The three pictures in the left row show the ES groups, and those in the right row show the non-ES groups. 

In the non-ES groups, four weeks after surgery, some newly formed bone evidently grew into the GGT construct (Figure 5(b)); eight weeks after surgery, the implantation site contained more new bone in the GGT construct (Figure 5(d)); twelve weeks after surgery, the amount of bone regeneration in the GGT construct exceeded that in the radiograph obtained after 8 weeks (Figure 5(f)). The three pictures also showed a U-shape trend in the new bone formation in the non-ES groups.

The images obtained four weeks after surgery displayed a larger amount of new bone formation in the ES groups than in the non-ES groups (Figure 5(a)). The radiograph obtained eight weeks after surgery showed the same pattern, and the newly formed bone accumulated on bilateral sides (Figure 5(c)). These two observations were more pronounced in the 12-week radiograph. The newly formed bone occupied a larger volume of the calvarial bone defect than in the 4-week and 8-week radiographs. The trend of the new bone to grow on bilateral sides was more obvious (Figure 5(e)). 

Table 1 shows the volume of newly formed bone for both the ES and non-ES groups for each implantation period. The volume of newly formed bone gradually increased with time. For each implant period, the volume of bone regeneration was obviously higher in the ES groups than in the non-ES groups. 

### 3.4. Histological Evaluation

**Figure 6:**
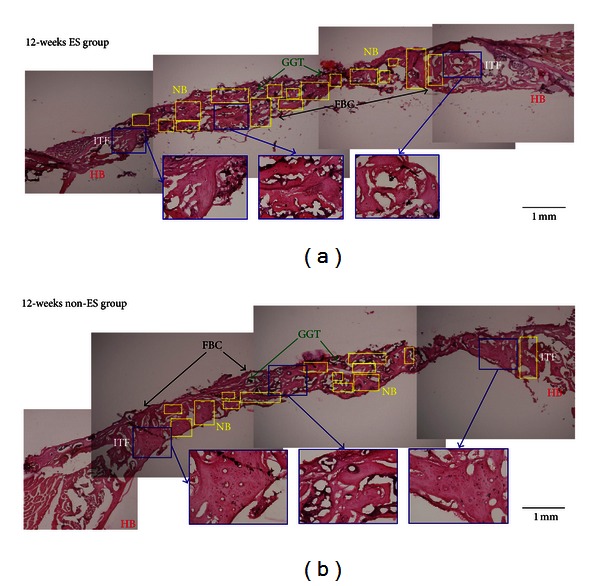
This figure shows transverse histological sections of calvarial defect 12 weeks after implantation with H&E stain. (a) displays the result of bone repair in the ES group. (b) shows the corresponding event for the control groups, in which non-ES was performed. The amount of bone regeneration in the rats is higher in the ES group than in the non-ES group (ITF: interface; NB: new bone; HB: host bone; FBC: foreign body capsule; original magnification: 40).

A histological evaluation was performed to compare the progress of restoration at the bone defect in the ES and non-ES groups.  Figure 6 shows the transverse sections with the H&E stain, demonstrating the difference in the growth rates of the new bone between the ES and non-ES groups. Twelve weeks after surgery, histological observation of the defective bone treated without ES indicated much new bone formation, as shown in Figure 6(b). However, compared with the control groups, the ES groups showed more new bone, indicating that ES could accelerate the restoration of defective bone (Figure 6(a)). The bone repair continued with time, and 12 weeks after implantation, the newly formed bone had replaced large amounts of the GGT composite. Substantially, more new bones were present in the defect after 12 weeks in the ES group than in the non-ES group (Figures 6(a) and 6(b)).

**Figure 7:**
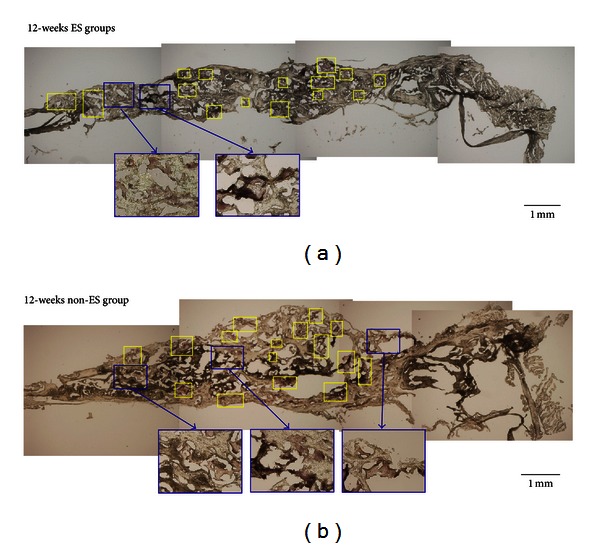
This figure shows longitudinal sections with ALP stain. The left side represents the far-end, in the direction of the cathode; the right side indicates the near-end, near the anode. For the ES group, the ALP accumulates on the left side after 12 weeks, as shown in (a); (b) illustrates the uniform distribution of ALP in the non-ES groups. The results indicate that osteoblasts are more active near the cathode in the ES groups.

**Figure 8:**
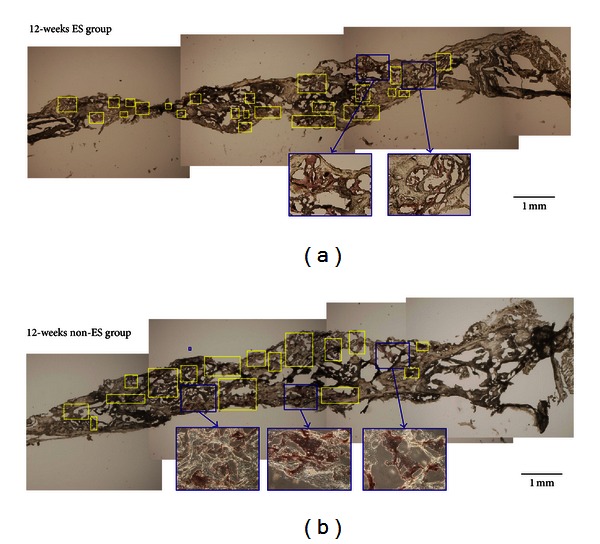
This figure shows longitudinal sections with TRAP stain. The left side represents the far-end, in the direction of the cathode; the right side indicates the near-end, near the anode. (a) shows the aggregation of TRAP on the right side in the ES group after 12 weeks. (b) illustrates the uniform distribution of TRAP in the non-ES groups. The results demonstrate that osteoclasts are vigorous near the anode in the ES groups.

Longitudinal sections with the ALP and TRAP stains identified the activity of osteoblasts and osteoclasts near the electrodes. In Figures 7 and 8, the left side represents the far-end, in the direction of the cathode on the head; the right side indicates the near-end, near the anode on the neck.  Figure 7 shows the slides with the ALP stain, and Figure 8 exhibits the TRAP stain. As shown in Figure 7(a), ALP accumulates on the left side in the ES groups, indicating that osteoblasts are more active near the cathode. In contrast, Figure 8(a) represents the aggregation of TRAP on the right side in the ES groups, demonstrating that osteoclasts are vigorous near the anode. When compared, Figures 7(b) and 8(b) illustrate the uniform distribution of ALP and TRAP in the non-ES groups.

## 4. Discussion

The GGT composite did not cause an obvious cytotoxic reaction in rabbits [7] or in the rats in this study. The radiographs from both X-ray and micro-CT showed the same trend. The new bone grew on bilateral sides of the electric current in the ES groups and grew in a U-shape in the non-ES groups. Osteogenesis depends on angiogenesis because blood vessels transport nutrients, oxygen, and stem cells. In the non-ES groups, the bottom of the U is near the heart, indicating that angiogenesis occurs from the heart to the far-end. Additionally, osteogenesis might rely on those vessels, resulting in the U-shaped formation. Previous researchers found that electric current stimulates bone regeneration at the cathode and, in contrast, bone resorption at the anode [22–24]. In the ES groups, more new bone accumulated on bilateral sides, extending to the far-end, in the direction of the cathode. Furthermore, much less new bone grew at the near-heart-end, around the anode. The mechanism may be related to the increasing pH level around the cathode; the pH is raised by the electric current and results in an increase in osteoblastic bone formation and a decrease in osteoclast bone resorption [25–27].

As shown with the data in Figure 4 and Table 1, the amount of new bone measured using micro-CT was much greater than the amount measured using X-ray during the early stage of examination. However, the data were similar in the late stage. These findings might result from the fact that the bone regeneration began around the interface between the host bone and the GGT composite, and the new bone expanded after filling the interface. Furthermore, Yao et al. also found that new bone grew in the centripetal direction [7]. Thus, we may underestimate the rate of bone repair when using 2D images from X-ray data. With this explanation, the information obtained from the 3D micro-CT data is considered more accurate. In addition, the images from micro-CT are obtained with live rats. Sacrificing the animals before examination is not necessary. Thus, serial data can be acquired from each individual rat, which could reduce the number of rats used in the experiment if micro-CT is the sole imaging technique in the study. 

The present study only investigated the appearance of bone regeneration using X-ray and micro-CT imaging. The hypothesis that the percutaneous electrical stimulation can accelerate the bone regeneration was confirmed in this study. However, the mechanism is still not very clear. The further study focused on the in-depth mechanism of ES which is undergoing.

## 5. Conclusion

 The study used a calvarial bone defect model to evaluate the effect of percutaneous electrical stimulation on bone regeneration. Radiographic analyses including X-ray and micro-CT show progressive bone healing with time. The bone repair rate is higher in the ES groups than in the non-ES groups. Additionally, the new bone grows on bilateral sides in the ES groups and accumulates in a U-shape in the non-ES groups. Histological evaluations with H&E stain also confirm the higher new bone formation rate in the ES groups. The slides with ALP and TRAP stains indicate that osteoblasts are more active near the cathode and that osteoclasts are more vigorous beside the anode. The results prove the thesis that percutaneous electrical stimulation can accelerate bone repair, and the bone regeneration is more active near the cathode than around the anode. Bone repair might rely on the activity of osteoblasts and osteoclasts by EA stimulation. 

## Figures and Tables

**Table 1:** The volume of new bone formation measured with micro-CT scan.

Implantation time	With/without ES	Volume (mm^3^) Mean ± SD
Four weeks*	With ES	16.70 ± 2.62
	Without ES	12.36 ± 2.24
Eight weeks*	With ES	22.03 ± 2.84
	Without ES	16.31 ± 2.49
Twelve weeks**	With ES	27.85 ± 2.16
	Without ES	18.29 ± 1.57

The volume of the newly-formed bone was determined in each implantation period (*n* = 4) (*P < 0.05, **P < 0.01).
